# First-trimester ultrasound measurements and maternal serum biomarkers as prognostic factors in monochorionic twins: a cohort study

**DOI:** 10.1186/s41512-019-0054-9

**Published:** 2019-05-09

**Authors:** Fiona L. Mackie, Rebecca Whittle, R. Katie Morris, Jon Hyett, Richard D. Riley, Mark D. Kilby

**Affiliations:** 1 Centre for Women’s and Newborn Health, Institute of Metabolism and Systems Research, University of Birmingham, Birmingham, B15 2TT UK; 2 Fetal Medicine Department, Birmingham Women’s and Children’s NHS Foundation Trust, Edgbaston, Birmingham, B15 2TG UK; 3 Centre for Prognosis Research, Research Institute for Primary Care & Health Sciences, Keele University, Keele, Staffordshire ST5 5BG UK; 4 Department of Women and Babies, Royal Alfred Hospital, University of Sydney, Sydney, Australia

**Keywords:** Monochorionic, PlGF, Prognostic factor, sFlt-1, Twin-twin transfusion syndrome, Twin pregnancy, AFP, Prediction

## Abstract

**Background:**

Monochorionic twin pregnancies are at high risk of adverse outcomes, but it is not possible to predict which pregnancies will develop complications. The aim of the study was to evaluate, in monochorionic twin pregnancies, whether first-trimester ultrasound (nuchal translucency [NT], crown-rump length [CRL]), and maternal serum biomarkers (alpha-fetoprotein [AFP], soluble fms-like tyrosine kinase-1 [sFlt-1] and placental growth factor [PlGF]), are prognostic factors for fetal adverse outcome composite, twin-twin transfusion syndrome (TTTS), growth restriction, and intrauterine fetal death (IUFD).

**Methods:**

A cohort study of 177 monochorionic diamniotic twin pregnancies. Independent prognostic ability of each factor was assessed by multivariable logistic regression, adjusting for standard prognostic factors. Factors were analysed as continuous data; thus, the reported ORs relate to either 1% change in NT or CRL inter-twin percentage discordance or one unit of measure in each serum biomarker.

**Results:**

The odds of the fetal adverse outcome composite were significantly associated with increased NT inter-twin percentage discordance (adjusted OR 1.03 [95% CI 1.01, 1.06]) and CRL inter-twin percentage discordance (adjusted OR 1.17 [95% CI 1.07, 1.29]). TTTS was significantly associated with increased NT discordance (adjusted OR 1.06 [95% CI 1.03, 1.10]) and decreased PlGF (adjusted OR 0.42 [95% CI 0.19, 0.93]). Antenatal growth restriction was significantly associated with increased CRL discordance (adjusted OR 1.20 [95% CI 1.08, 1.34]). Single and double IUFD were associated with decreased PlGF (adjusted OR 0.34 [95% CI 0.12, 0.98]) and (adjusted OR 0.18 [95%CI 0.05, 0.58]) respectively.

**Conclusion(s):**

This study has identified potential individual prognostic factors in the first trimester (fetal biometric and maternal serum biomarkers) that show promise but require further robust evaluation in a larger, prospective series of MC twin pregnancies, so that their usefulness both individually and in combination can be defined.

**Trial registration:**

ISRCTN 13114861 (retrospectively registered)

**Electronic supplementary material:**

The online version of this article (10.1186/s41512-019-0054-9) contains supplementary material, which is available to authorized users.

## Background

Multiple pregnancies are at increased risk of adverse outcome with twin perinatal mortality up to sevenfold higher compared to singleton pregnancies [1] with monochorionic diamniotic (MCDA) twins being at higher risk of pregnancy loss and morbidity compared to dichorionic twins [2]. These higher risks are secondary to the presence of inter-twin anastomoses joining the fetal circulations within the monochorionic (MC) placenta [3] and subsequently increasing the risk of twin-twin transfusion syndrome (TTTS) and fetal growth restriction (FGR) (either one or both fetuses affected) [4]. International guidelines recommend intensive antenatal surveillance to detect adverse outcomes complicating MC twins, principally TTTS and FGR. This involves regular monitoring via ultrasound scans from 16 weeks gestation at two weekly intervals to evaluate the liquor volume in each fetal sac, fetal biometry, and often fetoplacental Doppler assessment [5–8]. Such obstetric surveillance requires ultrasonographic expertise and health economic resources, it is time-consuming and targets all MC twins as a ‘high-risk population’. Additionally, this intensive surveillance may increase maternal anxiety and affect mental health. If it was possible to predict which MC twin pregnancies were at higher risk of developing complications, it would allow clinicians to stratify care, and those at higher risk could undergo more frequent surveillance, or be assessed earlier in a tertiary referral centre. This does have financial implications but may prevent fetal death as TTTS is a highly morbid and rapidly progressive condition, and even with current surveillance every 2 weeks, fetuses still die before FLA. Identifying high-risk pregnancies may enable the development of new therapies and prophylactic treatments that at present are not possible. Other benefits of prognostic factor research are that it informs adjustment in observational studies and stratification within randomisation of trials and provides the components of multivariable prognostic models [9, 10].

There has been interest in whether the risk of fetal disease of placental origin in singleton pregnancies could be predicted using fetal biometry combined with maternal biomarkers. The focus has been on circulating biochemical markers such as those utilised in first-trimester combined screening [11], i.e. pregnancy-associated plasma protein A (PAPP-A) and free β-human chorionic gonadotrophin (βhCG), but has also included alpha-fetoprotein (AFP) and factors related to angiogenesis and vasculogenesis such as placental growth factor (PlGF) and soluble fms-like tyrosine kinase-1 (sFlt-1). The evaluation of first-trimester ultrasound measurements in MCDA twins has noted that discordance in nuchal translucency (NT) and crown-rump length (CRL) have demonstrated relatively low sensitivity (~ 40%) and specificity (~ 60%) for the prediction of adverse pregnancy outcomes, particularly for TTTS [12–15].

Before developing multivariable risk prediction tools, it is essential to identify prognostic factors (predictors) that can be incorporated within the model. This requires prognostic factor research [9, 10]. In MCDA twins complicated by untreated TTTS, we have demonstrated that the second-trimester maternal serum concentrations of AFP and the ratio of sFlt-1 to PlGF were significantly higher in TTTS compared to gestationally matched uncomplicated MCDA twins [16, 17]; however, there has been little research on the prognostic ability of first-trimester maternal serum [18].

This paper examines the prognostic ability of first-trimester ultrasound biometry and maternal serum biomarkers for adverse outcome in MC twin pregnancies. We hypothesise that, in those with at higher risk, NT, CRL, AFP, and sFlt-1 will be increased, and PlGF will be decreased in the first trimester, preceding the clinical features associated with adverse outcome, and thus may be important prognostic factors.

## Methods

This work is part of a larger study which was registered in April 2016: ISRCTN 13114861 (www.isrctn.com/ISRCTN13114861). The protocol was published prior to analysis [19]. Since submitting the protocol for publication, a collaboration with the Royal Prince Alfred Hospital, Sydney, Australia, was formed to generate a multicentre, international cohort.

### Participants

All women with a MCDA twin pregnancy who had undergone first-trimester aneuploidy screening in the West Midlands and North Thames Regions, UK (October 2014 to September 2015), or at the Royal Prince Alfred Hospital, Sydney (June 2011 to April 2016), had maternal serum stored with consent at − 80 °C. Chorionicity was determined by first-trimester ultrasound noting a single placental mass, thin inter-twin membrane, and the presence of the ‘T’ sign [20]. Pregnancies were prospectively dated using the largest twin CRL between 45 and 84 mm [5]. Women had prenatal care at 29 different secondary and tertiary care maternity units. Pregnancies were not eligible for inclusion if they were concordant or discordant for structural fetal anomalies, monoamniotic, higher-order multiples, or suffered a fetal loss < 14 weeks. Pregnancies were cared for according to local and international guidelines. Prospectively recorded outcome data until hospital discharge were retrospectively collected from the hospital notes.

### Prognostic factor measurements

The biomarkers to be examined for their prognostic ability were identified a priori, i.e. pre-defined before data collection and analysis to avoid data dredging and selective reporting.

### Ultrasound measurements

NT and CRL were performed using standard practice (Fetal Anomaly Screening Programme [FASP] or Fetal Medicine Foundation [FMF] protocols with UK National External Quality Service [NEQAS] quality assessment) in women who consented to first-trimester aneuploidy screening [21, 22]. NT discordance (%) was calculated as the smallest NT subtracted from the largest NT, divided by the largest NT, and multiplied by 100. CRL discordance (%) was calculated as per NT discordance. These measurements were treated as continuous data within analyses, and so, no cut-offs were applied [23].

### Biomarker measurements

Three serum biomarkers (AFP, sFlt-1, PlGF) were measured on stored maternal serum samples that were initially analysed for β-hCG and PAPP-A as part of aneuploidy screening and were subsequently stored at − 80 °C (between 4 months and 5 years). The sFlt-1 and PlGF assays were performed in a single batch at the Biochemistry Department at University Hospital Birmingham, using the assay approved by the UK’s National Institute for Health and Care Excellence (NICE) for ruling out pre-eclampsia (Roche Diagnostics Limited, Sussex, UK) [24, 25]. The AFP assays were performed at the Biochemistry Department at Birmingham Women’s Hospital (Perkin Elmer Wallac Oy, Turku, Finland) after a single freeze-thaw cycle. The laboratories are CPA (UK) Ltd. accredited and are externally quality assessed by NEQAS and Down’s syndrome Quality Assurance Support Service (DQASS). The inter-assay and intra-assay coefficient of variation for measured serum AFP, sFlt-1, and PlGF were all < 5%.

### Adjustment for standard prognostic factors

Results were analysed on their original scale, as opposed to multiples of the median (MoMs), as all centres used the same method of measurement. Some researchers prefer using MoMs to deal with different measurements and case mix in each centre, but concerns have been raised [26]. Here, all centres used the same measurement method, and we adjusted for case mix via standard prognostic factors. Multivariable analyses were adjusted for standard clinical information considered to be standard prognostic factors: maternal BMI, age, smoking status, ethnicity, parity, and mode of conception (Table 1). The neonatal outcome was also adjusted for gestational age at delivery and steroid and antenatal magnesium sulphate administration. It was not possible to adjust for gestational age at blood sampling as gestational age was calculated based on CRL which is one of the potential prognostic factors under evaluation.

**Table 1:** Maternal characteristics used as adjustment factors

	Fetal composite (TTTS, TAPS, TOPS, IUGR, IUFD)	
	At least one fetal complication present (*n* = 94)	No fetal complication (*n* = 83)
Maternal age, mean (SD) years	30.73 (5.30)	29.99 (5.53)
Maternal BMI, mean (SD) kg/m^2^	24.71 (5.08)	25.05 (5.77)
Maternal smoking status, *n* (%)		
Never	64 (75.29)	63 (79.75)
Current smoker	8 (9.41)	4 (5.06)
Ex-smoker	13 (15.29)	12 (15.19)
Maternal ethnicity, *n* (%)		
White	60 (64.52)	52 (65.00)
Mixed	3 (3.23)	7 (8.75)
Oriental	11 (11.83)	11 (13.75)
South Asian	12 (12.90)	7 (8.75)
African-Caribbean	7 (7.53)	3 (3.75)
Parity, *n* (%)		
0	61 (64.89)	46 (55.42)
1	23 (24.47)	25 (30.12)
2	8 (8.51)	10 (12.05)
3	1 (1.06)	1 (1.20)
4	1 (1.06)	1 (1.20)
Assisted conception, *n* (%)	14 (15.22)	10 (12.50)
Gestational age at delivery, median (IQR)	34.36 (30.00, 36.43)	36.00 (35.00, 36.57)
Steroids administration	67 (71.28)	58 (70.73)
Magnesium sulphate administration	10 (10.64)	2 (2.44)

*BMI* body mass index, *IUFD* intrauterine fetal death, *IUGR* intrauterine growth restriction, *TAPS* twin anaemia polycythaemia sequence, *TOPS* twin oligohydramnios-polyhydramnios sequence, *TTTS* twin-twin transfusion syndrome

### Outcomes

There is no internationally agreed core outcome set for twin pregnancies; therefore, outcomes were formulated by our research team. The primary outcome of our study was a fetal adverse outcome composite defined as at least one of the following: TTTS, antenatally detected growth restriction, postnatally detected growth restriction, twin anaemia polycythaemia sequence (TAPS), twin oligohydramnios-polyhydramnios sequence (TOPS) or intrauterine fetal death (IUFD) (Additional file 1). We also examined individual complications as secondary outcomes:

- TTTS: defined and staged as per Quintero criteria [27]. Pregnancies affected by TTTS with concurrent growth restriction were not included in the antenatal or postnatally detected growth restriction groups
- Antenatally detected fetal growth restriction: abdominal circumference (AC) or estimated fetal weight (EFW) < 10th centile in either/both fetus(es) and/or growth discordance > 20% recorded at least twice over ≥ 2-week period
- Postnatally detected growth restriction: birthweight < 9th centile on the World Health Organization Growth Charts [28]
- IUFD: sub-classified as either single IUFD (sIUFD) or double IUFD (dIUFD). The pregnancy was considered a miscarriage if the pregnancy loss occurred at 14–24 weeks and a stillbirth if ≥ 24 weeks
- Spontaneous preterm birth (PTB): between 24 and 34 weeks gestation. Iatrogenic PTB delivery was not included
- Neonatal composite outcome: see Additional file 1
- Maternal composite outcome: see Additional file 1

### Statistical analysis

All analyses were performed using Stata/MP 14.0 (Stata Corporation, TX, USA). If > 5% data were missing (19% for maternal BMI and 7% for smoking status), multiple imputation was performed to replace the missing values using a chained equation approach, with predictive mean matching for continuous variables, based on the existing and potential prognostic factors and fetal adverse outcome composite outcome [29, 30]. Ten imputed datasets were created and results were combined across all datasets using Rubin’s rules to obtain final model estimates [31]. The neonatal outcome was missing for 14 babies, but the missing data were not imputed.

For each of the primary and secondary outcomes, univariable logistic regression models were fitted, calculating the unadjusted odds ratio (95% confidence interval) for the association between each potential prognostic factor and each outcome. Multivariable logistic regression models were then used to estimate the independent prognostic value (i.e. adjusted odds ratio (aOR) and its 95% confidence interval) of each potential prognostic factor after adjusting for standard prognostic characteristics defined a priori: maternal booking BMI, age, smoking status, ethnicity, parity, and mode of conception. The *p* value is also presented for the aOR. To gauge the potential increase in discrimination performance of a prognostic model that includes each potential prognostic factor in addition to existing factors, the change in apparent c-statistic (increase in area under the curve) for each outcome was calculated (i.e. difference in apparent c-statistic for models with standard characteristics including or excluding each factor). No adjustment for potential model overfitting was made during the calculation, as this was only for illustration of the potential impact of including the factors.

For outcomes relating to individual babies, a random intercept term at the level of the mother was included to account for clustering of multiple babies per mother. The clustering of babies within mothers for the outcome of IUFD was not able to be accounted for using a random effects model due to small numbers and minimal variation within the clusters which created convergence issues; hence, a fixed effect model was used. Clustering by hospital was also adjusted by putting a random effect on the intercept which allowed for heterogeneity in baseline risk across hospitals.

The serum biomarkers were log transformed as they were highly skewed. Continuous prognostic factors were included in the models as linear terms. Non-linear terms were considered by using fractional polynomials [32]; however, the best fitting models were those including a linear term only.

### Translating the prognostic effects into absolute risk

To translate how the absolute risk of developing the fetal adverse outcome might change across the distribution of each potential prognostic factor, two values for each potential prognostic factor were chosen a priori*.* The values for the ultrasound measurements were based on cut-offs used in existing literature for NT % discordance (0% and 20%) and CRL % discordance (0% and 10%). The values used for the maternal serum biomarkers were based on centiles in the study cohort: for AFP and sFlt-1, the 50th centile and 95th centile were used, and for PlGF, the 50th and 5th centile were used. For each of these values, absolute risk was estimated from the final model with other variables in the model given the mean values in the study cohort: 30 years old, BMI 25, non-smoker, Caucasian, nulliparous, and natural conception.

## Results

### Patient characteristics

One hundred seventy-seven MCDA twin pregnancies (354 fetuses) were identified and included in the study. The mean age of the participants was 30.4 years (SD 5.4), and mean BMI was 24.9 kg/m^2^ (SD 5.4). The majority of women had never smoked (77.4%), 60.5% were nulliparous, and most conceived spontaneously (86.0%). The maternal characteristics are summarised in Table 1.

The median gestational age at blood sampling was 12 + 6 weeks (IQR 12 + 3, 13 + 2). The median values of the potential prognostic factors in the cohort were inter-twin NT discordance 11.8% (IQR 6.6, 21.2), inter-twin CRL discordance 4.2% (IQR 1.7, 7.0), AFP 29.3 U/mL (IQR 23.4, 41.5), sFlt-1 2163 pg/mL (IQR 1645, 2945.5), and PlGF 60.5 pg/mL (IQR 40.9, 89.0).

Thirty-one percent experienced no adverse outcome and resulted in two healthy live births after 34 weeks of gestation (Table 2). Over half of the cohort (53.1%) had a pregnancy affected by at least one adverse outcome in the ‘fetal adverse outcome composite’. TTTS occurred in 13.0%, which presented at a median gestational age of 18 + 1 weeks (IQR 18, 24); the median Quintero stage was III, with 69% Quintero stage > II at presentation. Fifty-five percent of those with postnatally detected growth restriction were not identified antenatally. A substantial proportion of those with a sIUFD/dIUFD was complicated by TTTS: 63.6% (7/11) and 58.3% (7/12) respectively. Antenatal growth restriction affected 2/11 sIUFDs and 3/12 dIUFDs. There were two cases of TAPS, and no cases of TOPS.

**Table 2:** Number of events (per pregnancy (*n* = 177) unless otherwise stated)

	*N* (%)
Uncomplicated monochorionic diamniotic twin pregnancy, delivered > 34 weeks gestation	55/177 (31.07)
Fetal composite*	94/177 (53.11)
Twin-twin transfusion syndrome	23/177 (12.99)
Antenatal growth restriction	41/177 (23.16)
Antenatal growth restriction (per fetus)	73/354 (20.6)
Postnatal growth restriction	43/177 (24.29)
Postnatal growth restriction (per baby)	54/354 (15.25)
Intrauterine fetal death (single)	11/177 (6.21)
Intrauterine fetal death (double)	12/177 (6.78)
Maternal antenatal and postnatal composite **	46/177 (25.99)
Spontaneous preterm birth at 24–34 weeks	12/177 (6.78)
Neonatal composite **	91/340 (26.76)

*Fetal composite included at least one of the following: twin-twin transfusion syndrome, antenatally detected growth restriction, postnatally detected growth restriction, twin anaemia polycythaemia sequence, or intrauterine fetal death

**see Additional file 1 for full definitions of these composites

A quarter of pregnancies (26.0%) had at least one maternal complication, the most common complication being gestational diabetes (13.6%). There were severe complications of hypertensive disorders of pregnancy in 7.9%. Two cases were complicated by placental abruption (1.1%) and 7 (4.0%) pregnancies were complicated by massive obstetric haemorrhage. Three mothers developed renal or liver dysfunction (1.7%). In total, five (2.8%) needed admission to high-dependency or intensive care unit. There were no maternal deaths.

### Primary outcome

#### Fetal adverse outcome composite

There was a statistically significant unadjusted association between increased odds of fetal adverse outcome composite and increased inter-twin NT% discordance and increased CRL% discordance in the first trimester (Table 3). These associations remained after adjustment, demonstrating that these factors have independent prognostic value toward the fetal adverse outcome composite, with an estimated 3% (aOR 1.03 [95%CI 1.01, 1.06], *p* = 0.01) increase in the odds of experiencing an outcome in the fetal adverse outcome composite, for each 1% increase in NT discordance, and an estimated 17% (aOR 1.17 [95%CI 1.07, 1.29], *p* = 0.001) increase in the odds of experiencing an outcome in the fetal adverse outcome composite, for each 1% increase in CRL discordance. The baseline c-statistic (for the model including only standard characteristics) was 0.594, and the increase in the c-statistic when each prognostic factor was additionally included was quite high with the NT% discordance having an increase in the c-statistic of 0.045 and CRL% discordance 0.103. There were no statistically significant associations between any of the maternal serum biomarkers (AFP, PlGF, or sFLT-1) measured in the first trimester and subsequent fetal adverse outcome (as measured by the composite), though confidence intervals were wide.

**Table 3:** Association between potential prognostic factors and outcome (*n* = 177 pregnancies)

Potential prognostic factor	Unadjusted OR (95%CI)	Adjusted* OR (95%CI)	*p* value	Change in c-statistic^†^
Fetal adverse outcome composite				
NT (% discordance)	*1.03 (1.01, 1.05)*	*1.03 (1.01, 1.06)*	*0.011*	*0.045*
CRL (% discordance)	*1.16 (1.06, 1.27)*	*1.17 (1.07, 1.29)*	*0.001*	*0.103*
AFP	1.91 (0.93, 3.94)	2.08 (0.94, 4.59)	0.071	0.026
sFlt-1	1.12 (0.52, 2.40)	1.03 (0.42, 2.50)	0.950	< 0.001
PlGF	0.73 (0.44, 1.22)	0.65 (0.37, 1.13)	0.125	0.014
Twin-twin transfusion syndrome (TTTS)				
NT (% discordance)	*1.05 (1.02, 1.08)*	*1.06 (1.03, 1.10)*	*< 0.001*	*0.137*
CRL (% discordance)	1.07 (0.96, 1.20)	1.09 (0.97, 1.23)	0.161	0.032
AFP	*3.04 (1.05, 8.78)*	*3.24 (1.00, 10.48)*	*0.050*	*0.067*
sFlt-1	1.91 (0.62, 5.88)	1.64 (0.44, 6.03)	0.459	0.006
PlGF	*0.43 (0.20, 0.91)*	*0.42 (0.19, 0.93)*	*0.032*	*0.074*
Antenatal growth restriction				
NT (% discordance)	1.01 (0.99, 1.03)	1.01 (0.99, 1.04)	0.261	0.014
CRL (% discordance)	*1.17 (1.06, 1.30)*	*1.20 (1.08, 1.34)*	*0.001*	*0.119*
AFP	1.55 (0.67, 3.55)	2.10 (0.82, 5.40)	0.123	0.032
sFlt-1	1.25 (0.50, 3.13)	1.47 (0.49, 4.35)	0.491	0.011
PlGF	0.91 (0.50, 1.66)	0.88 (0.44, 1.76)	0.720	−0.001
Single intrauterine fetal death (sIUFD)				
NT (% discordance)	1.01 (0.98, 1.05)	1.02 (0.98, 1.06)	0.425	<−0.001
CRL (% discordance)	*1.17 (1.00, 1.36)*	*1.19 (1.01, 1.40)*	*0.035*	*0.085*
AFP	0.68 (0.16, 2.87)	0.80 (0.17, 3.90)	0.787	−0.004
sFlt-1	1.27 (0.27, 6.04)	1.79 (0.30, 10.64)	0.523	<−0.001
PlGF	*0.35 (0.13, 0.97)*	*0.34 (0.12, 0.98)*	*0.045*	*0.057*
Double intrauterine fetal death (dIUFD)				
NT (% discordance)	1.02 (0.98, 1.06)	1.02 (0.98, 1.06)	0.420	−0.005
CRL (% discordance)	1.06 (0.91, 1.24)	1.12 (0.94, 1.33)	0.221	0.019
AFP	1.33 (0.34, 5.21)	0.97 (0.18, 5.33)	0.970	<−0.001
sFlt-1	4.13 (0.92, 18.58)	8.21 (1.02, 66.24)	0.048	0.035
PlGF	*0.23 (0.08, 0.63)*	*0.18 (0.05, 0.58)*	*0.005*	*0.080*

The AFP, sFlt-1, and PlGF are log_e_ transformed

*Adjusted for maternal BMI, age, smoking status, ethnicity, parity, and mode of conception. sIUFD and dIUFD were only able to be adjusted for maternal BMI, age, ethnicity, and mode of conception. Italics denote significant associations. *P* value is related to the adjusted OR

^†^Change in c-statistic represents the additional prognostic value of each individual potential prognostic factor above the standard prognostic factors (maternal BMI, age, smoking status, ethnicity, parity, and mode of conception). The baseline c-statistics for the fetal adverse outcome composite is 0.594, TTTS is 0.617, antenatal growth restriction is 0.616, sIUFD is 0.625, and dIUFD is 0.783

### Secondary outcomes

#### Twin-twin transfusion syndrome (TTTS)

Increased first-trimester inter-twin NT% discordance was associated with development of TTTS after adjustment for previously standard prognostic factors (aOR 1.06 [95%CI 1.03, 1.10], *p* < 0.001) with a 6% increase in the odds of developing TTTS, for each 1% increase in NT discordance (Table 3). Increased maternal serum AFP measured in the first trimester was associated with the development of TTTS before adjustment (OR 3.04 [95%CI 1.05, 8.78]). After adjustment for previously identified prognostic factors, this was no longer statistically significant (aOR 3.24 [95%CI 1.00, 10.48], *p* = 0.050); however, the majority of the confidence interval suggests a prognostic effect, although with large uncertainty about the magnitude. Decreased maternal serum PlGF measured in the first trimester was associated with the development of TTTS before adjustment (OR 0.43 [95%CI 0.20, 0.91]), and this remained after adjustment for previously identified prognostic factors (aOR 0.42 [95%CI 0.19, 0.93], *p* = 0.03), demonstrating an estimated 42% increase in the odds of TTTS for each concentration unit decrease in log_e_-PlGF. The baseline c-statistic (for the model with only standard prognostic characteristics) was 0.617, and the increase in the c-statistic when each prognostic factor was included was quite high for NT (0.137), AFP (0.067), and PlGF (0.074), respectively.

#### Antenatally detected growth restriction

Increased first-trimester inter-twin CRL% discordance was an independent prognostic factor for antenatally detected growth restriction (aOR 1.20 [95%CI 1.08, 1.34], *p* = 0.001) (Table 3). No other first-trimester fetal ultrasound or maternal biomarker measurements were associated with antenatally detected growth restriction. The baseline c-statistic was 0.616 (for the model with only standard prognostic characteristics).

#### Intrauterine fetal death (IUFD)

The majority of the IUFDs were associated with TTTS and/or antenatally detected growth restriction (78%). It was not possible to separate spontaneous IUFDs from those associated with TTTS or growth restriction as the cohort numbers were too small. The only statistically significant maternal serum biomarker was decreased PlGF which was a prognostic factor for sIUFD (aOR 0.34 [95% CI 0.12, 0.98], *p* = 0.045) and dIUFD (aOR 0.18 [95% CI 0.05, 0.58], *p* = 0.005) (Table 3). No other prognostic factors were found in the ultrasound measurements or serum biomarkers for the other outcomes, including maternal and neonatal outcomes (Additional file 2). The baseline c-statistic was 0.625 and 0.783 for sIUFD and dIUFD respectively (for the model with only standard prognostic characteristics).

#### Translating the prognostic effects into absolute risk

The odds of developing the fetal adverse outcome based on two values for each potential prognostic factor were translated to absolute risks, to demonstrate how the estimated prognostic effect for each individual factor might change absolute risk predictions, if a prognostic model were developed in the future. Predicted outcome risk is shown for various values of each factor and for individuals whose other standard prognostic factors are set at the mean in the dataset (Table 4). No adjustment for potential model overfitting was made, as this was only for illustration of the potential impact of the included factors on predicted risks from a prognostic model.

**Table 4:** Predicted risk of developing a fetal adverse outcome according to potential prognostic factor measurements determined a priori

Potential prognostic factor measurement	Predicted risk*
NT (% discordance) 0%	0.446
NT (% discordance) 20%	0.604
CRL (% discordance) 0%	0.379
CRL (% discordance) 10%	0.750
AFP 50th centile (29.3 U/mL)	0.549
AFP 95th centile (54.7 U/mL)	0.658
PlGF 50th centile (60.4 pg/mL)	0.535
PlGF 5th centile (23.6 U/mL)	0.634
sFlt-1 50th centile (2169 pg/mL)	0.545
sFlt-1 95th centile (4089 pg/mL)	0.549

*Risk predictions obtained from the fitted multivariable model for values shown and using the mean values of the standard factors included in the model: 30 years old, BMI 25, non-smoker, Caucasian, nulliparous, and natural conception

## Discussion

### Main findings

Increasing percentage difference in NT and CRL was statistically significantly associated with our fetal adverse outcome composite, including after adjustment for standard prognostic factors defined by maternal variables. Increasing inter-twin CRL discordance was also statistically significantly associated with IUFD and antenatally detected growth restriction, whilst an increasing discordance in inter-twin NT was statistically significantly associated with the development of TTTS. When the association was considered at an individual level in clinical scenarios, there was a potential clinical utility of individual biomarkers.

### Strengths and limitations

Our study has the benefit of investigating the prognostic values of inter-twin NT and CRL percentage discordance as a continuous variable whereas other studies dichotomised the data using non-validated ‘cut-offs of abnormality’ which loses important information (often equivalent to throwing away one third of the data) [33].

When evaluating maternal serum biomarkers in our study, neither serum AFP, PlGF, nor sFlt-1 measured between 11 + 0 and 13 + 6 weeks was prognostic for the fetal adverse outcome composite. However, unadjusted logistic regression indicated that both AFP and PlGF were significantly associated with the development of TTTS. On adjusting for existing standard prognostic factor, the association persisted for PlGF and approached significance for AFP. These findings are slightly different to our previous findings that second-trimester maternal serum AFP and sFlt-1 to PlGF ratio are significantly elevated in MC twin pregnancies complicated by TTTS compared to uncomplicated MC twin pregnancies; however, this was when signs of TTTS were already apparent and the condition had been diagnosed [16, 17].

This study is the first that an association between first-trimester maternal serum AFP and PlGF and the adverse outcome has been noted, and it should be emphasised that this association is prior to the development of clinical TTTS. We did this using widely utilised and validated methodologies used in accredited biochemistry laboratories. In our cohort study, sIUFD and dIUFD occurred in pregnancies affected by TTTS in a substantial number of cases; thus, it is possible that the secondary outcome of IUFD was associated with the presence of TTTS. This is an interesting finding and it may be that PlGF could be used as a marker of severity of TTTS, although this does need further investigation and was not the aim of this study. The exact mechanisms for the development of TTTS are complex. The association between TTTS and decreased PlGF may be biologically plausible as PlGF is pro-vasculogenic and pro-angiogenic [34] and TTTS is related to the formation of inter-twin placental anastomoses. The borderline association with increased AFP warrants further investigation as it is a marker of placental function [35], and although we previously found a significant increase in second-trimester maternal serum from TTTS pregnancies [16], another study found no difference [36]. The limitations of this study are that it is retrospective and the cohort size is relatively small. For the maternal biomarkers, the confidence intervals surrounding the corresponding odds ratios are large, meaning the estimates are not very precise. Hence, this large variability needs to be investigated in further research with larger sample sizes, allowing the assessment of multiple prognostic factors in combination. This could enable the development of a prognostic model, depending on the predictive ability. The other important consideration is that although there is a call for universal definitions of outcome in MC twin pregnancies [37], this is not yet internationally agreed. This is particularly true for TTTS and antenatal fetal growth restriction in MC twins. Recently, a Delphi consensus was published to focus definitions and outcomes in fetal growth restriction in twins [38], but this requires validation. Thus, we chose fetal adverse outcome composite as an endpoint, as well as individual pathologies reported in MC twin pregnancies. Although we were as pragmatic as possible with the growth restriction definitions, different pathophysiological mechanisms [39] may be included as the sample size was too small to do further sub-group analysis, possibly explaining why biomarkers were not significant for the growth restriction outcomes. It may also be that “true” or “confirmed” growth restriction was not represented due to the discrepancy between antenatally and postnatally detected growth restriction not being concordant, although the growth restriction definitions were chosen as the antenatal measurements are those that guide clinicians management decisions. However, our analysis was performed according to robust prognostic methodology and has been reported in keeping with the REporting recommendations for tumor MARKer prognostic studies (REMARK) [40].

### Interpretation

The aim of this work was to evaluate the prognostic ability of individual first-trimester markers for adverse outcome in MC twins. For those markers where there was a statistically significant independent association with adverse outcome, calculating the absolute risk for each outcome using common values demonstrated that the markers may be useful clinically, although this depends on the change in management.

A recent systematic review by Stagnati [15] summarised the results of seven studies comprising 1087 MC twin pregnancies. These studies defined discordant NT in various ways: > 10%, > 20%, or a difference of > 0.5 or 0.6 mm. Of the MC twins, 128 developed TTTS, the only adverse outcome examined. The meta-analysis demonstrated that NT% discordance and NT> 95th centile had a prognostic association with the development of TTTS: positive likelihood ratio (LR+) 1.92 [95%CI 1.25, 2.96] and negative likelihood ratio (LR−) 0.65 [95% CI 0.50, 0.84] and LR+ 2.63 [95%CI, 1.51, 4.58] and LR− 0.85 [95% CI, 0.75, 0.96], respectively. However, overall the ‘pooled data’ demonstrated a low sensitivity (52.8% [95% CI 43.8, 61.7]; *I*^2^ = 48.7%) and specificity (72.5% [95% CI 61.7], 82.0; *I*^2^ = 84.3%) for detecting TTTS. Our work has examined the biomarkers as continuous variables, to ensure that no prognostic ability is lost by choosing an arbitrary threshold, but has demonstrated a similar association with adverse outcome but limited translation for individual biomarkers into a clinically useful predictive tool.

## Conclusions

Currently, there are no established prognostic models for predicting adverse outcome in MC twins. This study has identified potential individual prognostic factors in the first trimester (fetal biometric and maternal serum biomarkers) that show promise but require further robust evaluation in a larger, prospective series of MC twin pregnancies, so that their usefulness both individually and in combination can be defined [9]. When larger datasets are available, these markers could potentially be combined with standard prognostic variables to form a prognostic model ready for internal and external validation.

## Additional files

Additional file 1: Definition and justification of outcomes. (DOCX 19 kb)

Additional file 2: Additional analyses. (DOCX 13 kb)

## Abbreviations

AFP: Alpha-fetoprotein
aOR: Adjusted odds ratio
BMI: Body mass index
CI: Confidence interval
CRL: Crown-rump length
dIUFD: Double intrauterine fetal death
FASP: Fetal Anomaly Screening Programme
FMF: Fetal Medicine Foundation
IQR: Interquartile range
IUFD: Intrauterine fetal death
IUGR: Intrauterine growth restriction
MC: Monochorionic
MCDA: Monochorionic diamniotic
NEQAS: UK National External Quality Service
NICE: National Institute for Health and Care Excellence
NT: Nuchal translucency
OR: Odds ratio
PAPP-A: Pregnancy-associated plasma protein-A
PlGF: Placental growth factor
PTB: Preterm birth
REMARK: REporting recommendations for tumor MARKer prognostic studies
SD: Standard deviation
sFlt-1: Soluble fms-like tyrosine kinase-1
TAPS: Twin anaemia polycythaemia sequence
TOPS: Twin oligohydramnios-polyhydramnios sequence
TTTS: Twin-twin transfusion syndrome

## References

[1] Chitrit Y, Filidori M, Pons J-C, Duyme M, Papiernik E (1999). Perinatal mortality in twin pregnancies: a 3-year analysis in Seine Saint-Denis (France). Eur J Obstet Gynecol Reprod Biol.

[2] Hack KEA, Derks JB, Elias SG, Franx A, Roos EJ, Voerman SK, Bode CL, Koopman-Esseboom C, Visser GHA (2008). Increased perinatal mortality and morbidity in monochorionic versus dichorionic twin pregnancies: clinical implications of a large Dutch cohort study. BJOG.

[3] Moldenhauer J, Johnson M (2015). Diagnosis and management of complicated monochorionic twins. Clin Obstet Gynecol.

[4] Oepkes D, Sueters M (2017). Antenatal fetal surveillance in multiple pregnancies. Best Pract Res Clin Obstet Gynaecol.

[5] Khalil A, Rodgers M, Baschat A, Bhide A, Gratacós E, Hecher K, Kilby MD, Lewi L, Nicolaides KH, Oepkes D, Raine-Fenning N, Reed K, Salomon LJ, Sotiriadis A, Thilaganathan B, Ville Y (2016). ISUOG Practice Guidelines: role of ultrasound in twin pregnancy. Ultrasound Obstet Gynecol.

[6] ACOG (2016). Practice bulletin No. 169: multifetal gestations: twin, triplet, and higher-order multifetal pregnancies. Obstet Gynecol.

[7] NICE, Multiple pregnancy (2011). The management of twin and triplet pregnancies in the antenatal period. NICE clinical guideline.

[8] Kilby M, Bricker L. RCOG Green-top Guideline No. 51: management of monochorionic twin pregnancy. BJOG. 2016, p. e2-35 10.1111/1471-0528.14188 27862859

[9] Riley R, Hayden J, Steyerberg E, Moons K, Abrams K, Kyzas P, Malats N, Briggs A, Schroter S, Altman D, Hemingway H (2013). Prognosis research strategy (PROGRESS) 2: prognostic factor research. PLoS Med.

[10] Riley R, van der Windt D, Croft P, Moons K. Prognosis research in healthcare: concepts, methods and impact. Oxford: Oxford University Press; 2019.

[11] Gaccioli F, Aye ILMH, Sovio U, Charnock-Jones DS, Smith GCS. Screening for fetal growth restriction using fetal biometry combined with maternal biomarkers. Am J Obstet Gynecol. 2018;218(2S):S725–37. 10.1016/j.ajog.2017.12.002 29275822

[12] Kagan KO, Gazzoni A, Sepulveda-Gonzalez G, Sotiriadis A, Nicolaides KH (2007). Discordance in nuchal translucency thickness in the prediction of severe twin-to-twin transfusion syndrome. Ultrasound Obstet Gynecol.

[13] El Kateb A, Nasr B, Nassar M, Bernard JP, Ville Y (2007). First-trimester ultrasound examination and the outcome of monochorionic twin pregnancies. Prenat Diagn.

[14] Johansen M, Oldenburg A, Rosthøj S, Maxild J, Rode L, Tabor A (2014). Crown−rump length discordance in the first trimester: a predictor of adverse outcome in twin pregnancies?. Ultrasound Obstet Gynecol.

[15] Stagnati V, Zanardini C, Fichera A, Pagani G, Quintero RA, Bellocco R, Prefumo F (2017). Early prediction of twin-to-twin transfusion syndrome: systematic review and meta-analysis. Ultrasound Obstet Gynecol.

[16] Fox CE, Pretlove SJ, Chan BC, Mahony RT, Holder R, Kilby MD (2009). Maternal serum markers of placental damage in uncomplicated dichorionic and monochorionic pregnancies in comparison with monochorionic pregnancies complicated by severe twin-to-twin transfusion syndrome and the response to fetoscopic laser ablation. Eur J Obstet Gynecol Reprod Biol.

[17] Fox CE, Lash GE, Pretlove SJ, Chan BC, Holder R, Kilby MD (2010). Maternal plasma and amniotic fluid angiogenic factors and their receptors in monochorionic twin pregnancies complicated by twin-to-twin transfusion syndrome. Ultrasound Obstet Gynecol.

[18] Mackie FL, Hall M, Morris R, Kilby M. Early prognostic factors of outcomes in monochorionic twin pregnancy: systematic review and meta-analysis. Am J Obstet Gynecol. 2018;219(5):436–46. 10.1016/j.ajog.2018.05.008 29763608

[19] Mackie FL, Morris RK, Kilby MD. The prediction, diagnosis and management of complications in monochorionic twin pregnancies: the OMMIT (Optimal Management of Monochorionic Twins) study. BMC Pregnancy Childbirth. 2017;17:153. 10.1186/s12884-017-1335-3 PMC5446741 28549467

[20] Sepulveda W, Sebire N, Hughes K, Odibo A, Nicolaides K (1996). The lambda sign at 10–14 weeks of gestation as a predictor of chorionicity in twin pregnancies. Ultrasound Obstet Gynecol.

[21] FASP (2015). Fetal anomaly screening programme.

[22] FMF (2004). The 11–13+6 weeks scan.

[23] Royston P, Altman D, Sauerbrei W (2006). Dichotomizing continuous predictors in multiple regression: a bad idea. Stat Med.

[24] Schiettecatte J, Russcher H, Anckaert E, Mees M, Leeser B, Tirelli AS, Fiedler GM, Luthe H, Denk B, Smitz J (2010). Multicenter evaluation of the first automated Elecsys sFlt-1 and PlGF assays in normal pregnancies and preeclampsia. Clin Biochem.

[25] NICE (2016). PlGF-based testing to help diagnose suspected pre-eclampsia (triage PlGF test, Elecsys immunoassay sFlt-1/PlGF ratio, DELFIA Xpress PlGF 1–2-3 test, and BRAHMS sFlt-1 kryptor/BRAHMS PlGF plus Kryptor PE ratio).

[26] Bishop J, Dunstan F, Nix B, Reynolds T, Swift A (1993). All MoMs are not equal: some statistical properties associated with reporting results in the form of multiples of the median. Am J Hum Genet.

[27] Quintero R, Morales W, Allen M, Bornick O, Johnson P, Kruger M (1999). Staging of twin-twin transfusion syndrome. J Perinatol.

[28] RCPCH (2016). Early years - UK-WHO growth charts and resources.

[29] White I, Royston P, Wood A (2011). Multiple imputation using chained equations: issues and guidance for practice. Stat Med.

[30] Little R (1988). Missing-data adjustments in large surveys. J Bus Econ.

[31] Rubin D (1987). Multiple imputation for nonresponse in surveys.

[32] Sauerbrei W, Royston P (1999). Building multivariable prognostic and diagnostic models: transformation of the predictors by using fractional polynomials. J R Statist Soc A.

[33] Altman D, Royston P (2006). The cost of dichotomising continuous variables. BMJ.

[34] Carmeliet P, Moons L, Luttun A, Vincenti V, Compernolle V, De Mol M, Wu Y, Bono F, Dew L, Beck H (2001). Synergism between vascular endothelial growth factor and placental growth factor contributes to angiogenesis and plasma extravasation in pathological conditions. Nat Med.

[35] Mizejewski G (2001). Alpha-fetoprotein structure and function: relevance to isoforms, epitopes, and conformational variants. Exp Biol Med.

[36] Sermondade N, Dreux S, Oury JF, Muller F (2009). Second-trimester maternal serum screening for Down syndrome in twin-to-twin transfusion syndrome. Prenat Diagn.

[37] COMET (2016). Developing a core outcome set for research in multiple pregnancies, COMET Initiative.

[38] Khalil A, Beune I, Hecher K, Wynia K, Ganzevoort W, Reed K, Lewi L, Oepkes D, Gratacos E, Thilaganathan B, Gordijn S. Consensus definition and essential reporting parameters of selective fetal growth restriction in twin pregnancy: a Delphi procedure. Ultrasound Obstet Gynecol. 2019;53(1):47–54. 10.1002/uog.19013 29363848

[39] Lewi L, Gucciardo L, Huber A, Jani J, Van Mieghem T, Done E, Cannie M, Gratacos E, Diemert A, Hecher K, Lewi P, Deprest J (2008). Clinical outcome and placental characteristics of monochorionic diamniotic twin pairs with early- and late-onset discordant growth. Am J Obstet Gynecol.

[40] McShane L, Altman D, Sauerbrei W, Taube S, Gion M, Clark G. Reporting recommendations for tumor marker prognostic studies (REMARK). J Natl Cancer Inst. 2005;97. 10.1093/jnci/dji237 16106022

