# Supplementary material for: First-trimester ultrasound measurements and maternal serum biomarkers as prognostic factors in monochorionic twins: a cohort study
Source: Diagn Progn Res. 2019 May 9;3:9. doi: 10.1186/s41512-019-0054-9 (PMC6507122; doi:10.1186/s41512-019-0054-9)
Supplement: Supplementary file 1 — Definition and justification of outcomes. (DOCX 19 kb) [file 41512_2019_54_MOESM1_ESM.docx]

**Additional file 1 Definition and justification of outcomes**

The primary outcome was a composite of adverse fetal events defined as the presence of at least one of the following: TTTS, antenatally-detected growth restriction, postnatally-detected growth restriction, twin anemia polycythemia sequence (TAPS), or IUFD.

Secondary outcomes and their definitions are listed below.

1. TTTS: polyhydramnios (>8cm in the deepest vertical pocket of the recipient at <20 weeks of gestation or >10cm from >20 weeks of gestation) in combination with oligohydramnios in the donor (<2 cm deepest vertical pool depth irrespective of gestation) in same sex twins diagnosed as MCDA twins in the first trimester. It was staged as per Quintero staging criteria (22). Pregnancies affected by TTTS with concurrent growth restriction were not included in the antenatal or postnatally-detected growth restriction groups as the etiology of the growth restriction is different and would make the growth restriction group heterogeneous.
2. Antenatally-detected growth restriction: abdominal circumference [AC] or estimated fetal weight [EFW] <10^th^ centile in either/both fetus(es), and/or growth discordance >20% recorded at least twice over ≥2 week period. As a result of different measures and definitions of twin growth, for which there is at present no international consensus, the term ‘growth restriction’ was used to denote a twin which demonstrated pathologically abnormal growth. It was believed to be important to use the different measures, as even within UK units, despite the Royal College of Obstetricians and Gynaecologists (RCOG) Green-top guidelines for the Management of Monochorionic Twin Pregnancy (33), the measurements and definitions used to make clinical decisions were different. For the primary outcome of the fetal adverse outcome composite, there was no differentiation between antenatal and postnatal growth problems. This was because the reported accuracy of predicting fetal growth restriction on ultrasound using Hadlock’s formula in twin pregnancies has a sensitivity of 70.1% (95%CI 62.2-77.1), specificity of 86.4% (95%CI 82.5-89.6), positive predictive value of 67.9% (95%CI 60.1-75.0) and negative predictive value of 87.5% (95%CI 83.7-90.7) therefore by including a postnatal measure of growth restriction this would allow the inclusion of pathologically growth restricted babies who were missed on ultrasound scan, thus allowing complete reporting of pathology (34). If viewed pragmatically, it was felt that if a factor was able to predict which pregnancies would result in a low birthweight (<9^th^ centile), antenatally this pregnancy would be managed differently to a pregnancy with no signs of abnormal growth. However the ‘growth restriction’ group was also divided into fetal and neonatal outcomes to assess if the inclusion of birthweight in the composite made a substantial difference. Pregnancies were not considered to have a growth problem if there was only one abnormal ultrasound scan, and the subsequent scans were normal. When growth was examined as an individual outcome, it was divided into antenatal and postnatal growth problems. From both sub-groups pregnancies with TTTS and TAPS were excluded as the abnormal growth seen is these conditions is thought to be linked to the pathology of TTTS or TAPS and thus has a different etiology to those with solely growth problems. Twins with an IUFD were also excluded if the IUFD was not preceded by evidence of growth restriction in the pregnancy. By including these patients it would make the growth group heterogeneous. Included in the ‘antenatally-detected growth restriction’ group are:

- Small for gestational age (SGA) fetus. The definition of SGA was an estimated fetal weight (EFW) <10^th^ centile in English cohort, and an abdominal circumference (AC) <10^th^ centile in the Australian cohort. This was based on the growth chart each unit used as when these pregnancies were being cared for, and clinical decisions were being made, this was not on a specific and validated growth chart for twin pregnancies. Different definitions were required because of differing guidance in the two countries for clinical decisions.
- Intrauterine growth restriction (IUGR). As per SGA but with abnormal umbilical artery Dopplers. This may occur in one (see selective IUGR below) or both of the twins.
- Selective intrauterine growth restriction (sIUGR). One twin EFW <10^th^ centile or AC <10^th^ centile and with abnormal umbilical artery Doppler as classified by Gratacós et al. (35) as below.
- Type I positive end-diastolic flow (EDF) in the umbilical artery
- Type II persistently absent or reversed end-diastolic flow (AREDF)
- Type III intermittent absent or reversed end-diastolic flow (iAREDF) in the absence of fetal breathing.
- Inter-twin growth discordance. Antenatal growth discordance was defined as >20% difference in the EFW using Hadlock’s formula. This was calculated by subtracting the weight of the smallest twin from the biggest twin, then dividing by the weight of the biggest twin, and multiplying by 100.

1. Postnatally-detected growth restriction: birthweight <9^th^ centile World Health Organization charts. In the ‘postnatally-detected growth restriction’ group are fetuses with a birthweight <9^th^ centile on the WHO growth chart (0-2 years or preterm birth charts depending on gestation at delivery) (23). Although these charts were designed for singletons, no specific validated charts for twin pregnancies exist at present. It was not possible to use a specific weight cut-off for neonatal unit (NNU) admission as this is different at each NNU. Birthweight discordance was not included as the implications of this are not known, and it is not used clinically postnatally. For pregnancies in which only one twin had a birthweight <9^th^ centile and abnormal growth was not detected antenatally, we had to rely on consistency in the naming of twin 1 and twin 2 antenatally and postnatally.
2. IUFD: fetal death after 14 weeks gestation. This may be a single IUFD (sIUFD) whereby one twin died or a double IUFD (dIUFD whereby both twins died. The pregnancy was considered a miscarriage if the pregnancy loss occurred at 14-24 weeks, and a stillbirth if the loss occurred at ≥24 weeks. Pregnancies with a loss <14 weeks gestation were not eligible for inclusion in the study as the first trimester ultrasound scan and biomarkers are measured at this time, and the pathology and consequences of a loss at <14 weeks gestation is different to that of a loss >14 weeks gestation.
3. Spontaneous preterm birth (PTB): if it occurred between 24-34 weeks gestation. Any births less than 34 weeks were sub-classified as spontaneous or iatrogenic as it would be clinically most useful if there were factors to predict spontaneous PTB, whereas iatrogenic PTB is more reflective of disease severity as it is a treatment rather than a pathological process. Only spontaneous PTBs were included in this outcome.
4. Neonatal composite outcome: neonatal death, respiratory distress syndrome, assisted ventilation (Continuous positive airway pressure [CPAP] or endotracheal [ET] tube) for >24 hours, intraventricular hemorrhage or other brain injury, necrotizing enterocolitis, neonatal encephalopathy, chronic lung disease, severe jaundice requiring phototherapy, severe infection e.g. septicemia, meningitis, exchange transfusion, cardiac impairment, neurological impairment.
5. Maternal morbidity composite outcome: gestational diabetes mellitus, severe infection, hypertensive disorders (pregnancy induced hypertension requiring medication, pre-eclampsia, eclampsia or HELLP syndrome), placental abruption, venous thromboembolism, disseminated intravascular coagulopathy, High-Dependency or Intensive Care Unit admission, cerebrovascular event, renal or liver failure, pulmonary edema, massive obstetric hemorrhage (>2L EBL), acute fatty liver.
