# Supplementary material for: First-trimester ultrasound measurements and maternal serum biomarkers as prognostic factors in monochorionic twins: a cohort study
Source: Diagn Progn Res. 2019 May 9;3:9. doi: 10.1186/s41512-019-0054-9 (PMC6507122; doi:10.1186/s41512-019-0054-9)
Supplement: Supplementary file 2 — Additional analyses. (DOCX 13 kb) [file 41512_2019_54_MOESM2_ESM.docx]

**Additional file 2 Additional analyses**

**Antenatally-detected growth restriction per fetus (n=354 fetuses)**

|  | **Unadjusted**  ***OR (95% CI)*** | **Adjusted***  ***OR (95% CI)*** |
| --- | --- | --- |
| NT (mm) | 0.62 (0.15, 2.56) | 0.67 (0.13, 3.35) |
| CRL (mm) | 1.12 (0.99, 1.27) | 1.11 (0.97, 1.26) |

* Adjusted maternal BMI, age, smoking status, ethnicity, parity and mode of conception.

**Postnatally-detected growth restriction (n=177 pregnancies)**

|  | **Unadjusted**  ***OR (95% CI)*** | **Adjusted***  ***OR (95% CI)*** |
| --- | --- | --- |
| NT (% discordance) | 1.01 (0.98, 1.03) | 1.01 (0.98, 1.03) |
| CRL (% discordance) | 1.05 (0.95, 1.15) | 1.04 (0.94, 1.15) |
| AFP | 0.88 (0.39, 1.98) | 0.88 (0.35, 2.20) |
| sFlt-1 | 0.63 (0.25, 1.59) | 0.65 (0.22, 1.88) |
| PlGF | 1.55 (0.84, 2.85) | 1.62 (0.80, 3.29) |

* Adjusted for maternal BMI, age, smoking status, ethnicity, parity and mode of conception.

**Postnatally-detected growth restriction per baby (n=354 fetuses)**

|  | **Unadjusted**  ***OR (95% CI)*** | **Adjusted***  ***OR (95% CI)*** |
| --- | --- | --- |
| NT (mm) | 0.81 (0.39, 1.68) | 0.75 (0.36, 1.55) |
| CRL (mm) | 0.96 (0.90, 1.01) | 0.97 (0.91, 1.02) |

* Adjusted for maternal BMI, age, smoking status, ethnicity, parity and mode of conception.

**Intrauterine fetal death per fetus (n=354 fetuses)**

|  | **Unadjusted**  ***OR (95% CI)*** | **Adjusted***  ***OR (95% CI)*** |
| --- | --- | --- |
| NT (mm) | 1.46 (0.35, 6.08) | 1.20 (0.62, 2.30) |
| CRL (mm) | 0.94 (0.81, 1.08) | 0.99 (0.95, 1.03) |

* Adjusted for maternal BMI, age, smoking status, ethnicity, parity and mode of conception. Logistic regression used for adjusted model due to convergence issues

**Spontaneous preterm birth (n=177 pregnancies)**

|  | **Unadjusted**  ***OR (95% CI)*** | **Adjusted***  ***OR (95% CI)*** |
| --- | --- | --- |
| NT (% discordance) | 1.00 (0.64, 1.04) | 0.99 (0.95, 1.04) |
| CRL (% discordance) | 0.93 (0.77, 1.11) | 0.92 (0.76, 1.11) |
| AFP | 0.96 (0.24, 3.81) | 0.76 (0.15, 3.80) |
| sFlt-1 | 0.38 (0.08, 1.84) | 0.30 (0.05, 1.90) |
| PlGF | 0.69 (0.26, 1.82) | 0.70 (0.25, 1.98) |

* Adjusted for maternal BMI, age, ethnicity, parity and magnesium sulphate.

**Neonatal composite outcome per baby (n=340 babies)**

|  | **Unadjusted**  ***OR (95% CI)*** | **Adjusted***  ***OR (95% CI)*** |
| --- | --- | --- |
| NT (mm) | 0.93 (0.30, 2.89) | 1.07 (0.33, 3.50) |
| CRL (mm) | 0.99 (0.91, 1.08) | 1.00 (0.91, 1.09) |

* Adjusted for maternal BMI, age, smoking status, ethnicity, parity, mode of conception, gestation at delivery, administration of steroids, and magnesium sulphate. 14 neonatal outcomes were missing, and were not imputed.

**Maternal antenatal and postnatal composite outcome (n=177 pregnancies)**

|  | **Unadjusted**  ***OR (95% CI)*** | **Adjusted***  ***OR (95% CI)*** |
| --- | --- | --- |
| NT (% discordance) | 1.01 (0.99, 1.03) | 1.01 (0.98, 1.03) |
| CRL (% discordance) | 0.96 (0.87, 1.06) | 0.97 (0.87, 1.07) |
| AFP | 0.56 (0.25, 1.26) | 0.55 (0.21, 1.42) |
| sFlt-1 | 1.36 (0.57, 3.27) | 1.26 (0.44, 3.58) |
| PlGF | 0.78 (0.44, 1.39) | 0.70 (0.34, 1.42) |

* Adjusted for maternal BMI, age, smoking status, ethnicity, parity and mode of conception
